# The Effect of Lean-Seafood and Non-Seafood Diets on Fasting and Postprandial Serum Metabolites and Lipid Species: Results from a Randomized Crossover Intervention Study in Healthy Adults

**DOI:** 10.3390/nu10050598

**Published:** 2018-05-11

**Authors:** Mette Schmedes, Claudia Balderas, Eli Kristin Aadland, Hélène Jacques, Charles Lavigne, Ingvild Eide Graff, Øyvin Eng, Asle Holthe, Gunnar Mellgren, Jette Feveile Young, Ulrik Kræmer Sundekilde, Bjørn Liaset, Hanne Christine Bertram

**Affiliations:** 1 Department of Food Science, Aarhus University, 8000 Aarhus, Denmark; mette.schmedes@food.au.dk (M.S.); jettef.young@food.au.dk (J.F.Y.); uksundekilde@food.au.dk (U.K.S.); 2 Department of Chemistry, Centre for Analysis and Synthesis, Lund University, 221 00 Lund, Sweden; c.balderas@ictan.csic.es; 3 Institute of Food Science, Technology and Nutrition (ICTAN), Spanish National Research Council (CSIC), ES-28040 Madrid, Spain; 4 Institute of Marine Research, 5817 Bergen, Norway; Eli.Kristin.Aadland@hvl.no (E.K.A.); charles.lavigne@videotron.ca (C.L.); ingvild.graff@uni.no (I.E.G.); Bjorn.Liaset@hi.no (B.L.); 5 Faculty of Education, Western Norway University of Applied Sciences, 5063 Bergen, Norway; Asle.Holthe@hvl.no; 6 School of Nutrition, Université Laval, Laval, G1V 0A6 QC, Canada; Helene.Jacques@fsaa.ulaval.ca; 7 Hormone Laboratory, Haukeland University Hospital, 5021 Bergen, Norway; oyvin_eng@hotmail.com (Ø.E.); gunnar.mellgren@med.uib.no (G.M.); 8 Department of Clinical Science, University of Bergen, 5020 Bergen, Norway

**Keywords:** postprandial, seafood protein, metabolism, TMAO

## Abstract

The metabolic effects associated with intake of different dietary protein sources are not well characterized. We aimed to elucidate how two diets that varied in main protein sources affected the fasting and postprandial serum metabolites and lipid species. In a randomized controlled trial with crossover design, healthy adults (*n* = 20) underwent a 4-week intervention with two balanced diets that varied mainly in protein source (lean-seafood versus non-seafood proteins). Nuclear magnetic resonance spectroscopy and liquid chromatography-mass spectrometry analyses were applied to examine the effects of the two diets on serum metabolites. In the fasting state, the lean-seafood diet period, as opposed to the non-seafood diet period, significantly decreased the serum levels of isoleucine and valine, and during the postprandial state, a decreased level of lactate and increased levels of citrate and trimethylamine *N*-oxide were observed. The non-seafood diet significantly increased the fasting level of 26 lipid species including ceramides 18:1/14:0 and 18:1/23:0 and lysophosphatidylcholines 20:4 and 22:5, as compared to the lean-seafood diet. Thus, the lean-seafood diet decreased circulating isoleucine and valine levels, whereas the non-seafood diet elevated the levels of certain ceramides, metabolites that are associated with insulin-resistance.

## 1. Introduction

The postprandial response to a dietary exposure is complex and involves metabolic switches in several organs like liver, muscle and adipose tissue in order to handle the short-term disturbance and restore homeostasis. In fact, it has been proposed that “health” should not just be defined as “the absence of disease”, but is more precisely described as “resilience of homeostatic control”, i.e., the ability to manage and restore upon daily disturbances [1]. In association with this, Pellis et al. demonstrated in a dietary intervention study with crossover design that by examining the postprandial response to a postprandial challenge test, additional metabolic changes related to the dietary intervention were discovered that was not observed in the fasting state [2]. Furthermore, the resultant postprandial hyperglycemic state that follows meal consumption has been shown to better predict future mortality from cardiovascular diseases (CVD) compared to fasting glucose in both diabetic and normoglycemic individuals [3], and postprandial triacylglycerol (TAG) levels have been associated with future cardiovascular events [4]. Thus, the examination of the postprandial response to dietary exposures is of importance as it is anticipated to reflect the physiological and biochemical robustness to handle perturbations of the fasting state (homeostasis) related to intake of a diet and thus might reveal multiple aspects of health that are not apparent when studying only the fasting state. 

Health status of a biological system might be reflected in the pattern of metabolites [5] and may provide insight into alterations in glucose, protein and lipid metabolism associated with metabolic disorders. Analysis of the blood metabolome has revealed an association between branched-chain and aromatic amino acids (BCAA and AAA) and future diabetes [6], and recently, a metabolic profile characterized by elevated plasma levels of isoleucine, valine, lysine and tryptophan was observed in obese adults with non-alcoholic fatty liver disease NAFLD [7]. These findings suggest a link between changes in circulating BCAA and metabolic dysfunction. However, limited data are currently available examining the effect of diet on circulating levels of BCAA.

Epidemiologic studies examining the effects of dietary macronutrients on risk factors for CVD have primarily been focused on manipulating the dietary content of carbohydrate and fat without changing the protein content [8,9,10]. However, evidence from cohort studies has revealed a role of dietary protein of both animal and plant origin to influence the risk of CVD [11,12,13]. In fact, the type of dietary protein has been associated with differential effects on CVD risk [14,15]. Fish consumption has been associated with CVD protective effects and decreased CVD mortality [16,17], and particularly the beneficial effects of omega-3 fatty acids, which are abundantly present in fatty fish, have been investigated [18]. However, intake of lean-fish, which contain relatively low amounts of omega-3 fatty acids, has also been associated with beneficial effects on lipid metabolism [19], and on insulin sensitivity and the risk of type 2 diabetes [20,21]. Furthermore, in a recent Norwegian epidemiological study, lean-fish consumption once a week or more was associated with decreased postprandial TAG and increased high-density lipoprotein cholesterol (HDL-C) levels, and in men a decreased waist circumference and blood pressure were identified [22]. Thus, current evidence suggests a beneficial effect of lean fish consumption on CVD risk factors. Still, few data are available on the effect of lean fish and seafood on fasting and postprandial serum metabolites and lipid species in healthy humans under standardized and controlled dietary conditions. 

Previously we showed that lean-seafood and non-seafood protein sources differentially modulated risk factors for CVD [23] and for type 2 diabetes [24] as well as differently influenced changes in the metabolism and the gut microbiota as reflected by differences in the urinary metabolome and in the gut microbiome, respectively [25,26]. In this secondary outcome study, we aimed to investigate the effects of two diets with lean-seafood and non-seafood protein sources under standardized and controlled dietary conditions on serum metabolite changes by combining NMR spectroscopy and LC-MS analyses on fasting and postprandial serum samples. 

## 2. Materials and Methods 

### 2.1. Study Design 

The experimental design has been described by Aadland et al. [23]. Briefly, as outlined previously [25], a randomized, controlled trial with crossover design including two 4-week experimental periods separated by a 5-week washout period was conducted (Figure S1). Prior to each experimental period, participants followed a prudent diet formulated in accordance with the Norwegian nutrition recommendations for three weeks (run-in period). After the first 3-week run-in period half of the subjects were randomly assigned to the lean-seafood diet with lunch- and dinner meals including cod, pollack, saithe and scallops and the other half of the subjects to a non-seafood diet containing chicken, lean beef, turkey, pork, egg, milk and milk products. At the first and last day of each intervention period the subjects received a defined test meal with postprandial blood sampling. At the test day, subjects were resting and allowed to drink water only during the 6 h after ingestion of the test meal.

### 2.2. Subjects

The recruitment of human subjects and the participants’ physical and clinical characteristics has been described by Aadland et al. [23]. Briefly, as summarized previously [25], a total of 148 female and male subjects (aged 18–65) were recruited from the area of Bergen, Norway, of which healthy subjects, assessed by medical examination and fasting blood analysis, were included in the study. Twenty-seven subjects started the study, 20 subjects completed period 1 and 19 subjects (7 men and 12 women) completed the study. The sample size required to detect changes in circulating VLDL triacylglycerol was calculated by Aadland et al. [23] and a minimum of 16 subjects was needed to detect a treatment difference of ~25–30% in VLDL triacylglycerol at a probability level inferior to 0.05 and a power level corresponding to 80%. Briefly, the subjects were characterized by an average age (year) of 50.6 ± 3.4, body mass index (kg/m^2^) of 25.6 ± 0.7, blood pressure (mm Hg, systolic/diastolic) of 127.3 ± 2.1/76.6 ± 2.1, LDL (mmol/L) of 3.6 ± 0.2, HDL (mmol/L) of 1.7 ± 0.1, total cholesterol (mmol/L) of 5.3 ± 0.2, fasting triacylglycerol (mmol/L) of 1.0 ± 0.1, fasting glucose (mmol/L) of 5.1 ± 0.1 and fasting insulin (pmol/L) of 41 ± 4. Each participant signed written informed consent and the study protocol was approved by the Regional Committees for Medical and Health Research Ethics of Western Norway (Reference # 2012/1084). The study was registered at www.ClinicalTrials.gov (NCT01708681). 

### 2.3. Diets

The two experimental diets were given as 7-day rotating menus as described by Aadland et al. [23]. Briefly, as given previously [25], the diets were balanced with equivalent amounts of dietary fiber, carbohydrates (52% of energy), protein (19% of energy), lipids (29% of energy), monounsaturated, polyunsaturated and saturated fatty acids. To balance for the endogenous marine n-3 fatty acids present in the lean-seafood diets, cod liver oil was added to all non-seafood dinner meals prior to serving [23]. The protein intake from the experimental protein sources corresponded to 60% of total protein in both diets. The remaining dietary proteins were of vegetable and cereal origins. The mean nutritional, the amino acid and the fatty acid composition of the 7 days menus and the test meals have previously been described by Aadland et al. [23]. Subjects consumed their breakfasts, evening meal and snacks at home from an approved food list adjusted to their experimental diet and energy level. Subjects were instructed not to consume any food besides the provided prepared meals they were given, or the amount and type of foods included in the breakfast, evening meal and snack lists. In the beginning and end of each experimental period a test meal was served to the subjects prior to the postprandial blood sampling. 

### 2.4. Serum Sampling and Analyses

Blood sampling were performed at the first and last day of each experimental period. After an overnight fast, a fasting blood sample (−15 min, baseline) was withdrawn from the antecubital vein. The subjects ingested the test meal within 15 min, and postprandial blood samples were taken immediately after (0 min) and at 30, 60, 120, 240, and 360 min after completion of the test meal. The blood samples were placed at room temperature to allow blood coagulation followed by centrifugation at 2500× *g* for 5 min and stored at −80 °C until analysis.

### 2.5. Metabolite Profiling

#### 2.5.1. NMR Spectroscopy

Prior to analysis, the serum samples were thawed at room temperature, vortexed for 30 s, centrifuged (10,000× *g*, 5 min, 4 °C) and the supernatant was mixed with 300 µL D_2_O in a 5 mm NMR tube. Serum ^1^H NMR spectra were obtained at 310 K on a Bruker Avance III 600 spectrometer operating at a ^1^H NMR frequency of 600.13 MHz and equipped with a 5 mm TXI probe (Bruker BioSpin, Rheinstetten, Germany). One-dimensional spectra were acquired using a single 90° pulse experiment with a relaxation delay of 3 s and a spin-echo delay of 1 ms. The Carr–Purcell–Maiboom-Gill (CPMG) sequence was applied to attenuate broad signals from proteins and lipoproteins. Water suppression was achieved by irradiating the water peak during the relaxation delay, and a total of 64 scans were collected into 32 K data points spanning a spectral width of 17.36 ppm. An exponential window function with a line-broadening factor of 0.3 Hz was applied to the FID before Fourier transformation. The ^1^H NMR spectra were phase and baseline corrected manually using Topspin 3.0 (Bruker Biospin), and referenced to the α-glucose signal at 5.23 ppm. The assigned NMR peaks were validated using two-dimensional heteronuclear single quantum coherence experiments on a pooled blood sample. 

#### 2.5.2. Liquid Chromatogram-Mass Spectrometry Lipidomics

##### Chemicals

Ultrapure water was used to prepare all aqueous solutions (Milli-Q purification system). Mass spectrometry grade isopropanol, acetonitrile and ammonium formate and high-performance liquid chromatography (HPLC) grade methanol and methyl-tert-butyl ether (MTBE) was used for liquid chromatography.

##### Lipid Extraction

Serum lipids were extracted by MTBE using a slightly adapted protocol from Matyash et al. [27]. Briefly, 40 μL of serum was mixed with 150 μL of methanol. The sample was vortex mixed for 30 s, and 750 μL of MTBE was added followed by mixing during 10 min. After addition of 100 μL of MilliQ water the sample was mixed 30 s and centrifuged at 13,000× rpm for 10 min at 4 °C. The upper organic phase was transferred to a clean tube and evaporated to dryness at 35 °C in a vacuum evaporator for 2 h. Prior to analysis, the sample was reconstituted in 50 µL isopropanol-acetonitrile (90:10, *v*/*v*), vortexed for 10 s and transferred to a HPLC vial with insert and 4 μL was injected into the UPLC system for positive and negative ion mode analyses. Quality control samples were prepared by pooling 2 uL of each sample and were analyzed frequently to provide representative mean study samples containing all the metabolites that will be detected during the analysis, and to check reproducibility and stability of the analytical methodology.

##### Ultrahigh Performance Liquid Chromatography (UPLC) Conditions

The lipidomics analyses were performed using an ACQUITY UPLC system coupled to a XEVO-G2 Q-TOF (quadrupole time of flight) mass spectrometer (Waters Corporation, Milford, MA, USA) with electrospray ionization (ESI) source and an autosampler maintained at 4 °C. 

Separation of lipids was performed on a Acquity UPLC CSH C18 column (100 × 2.1 mm, 1.7 μm, Waters) coupled to an Acquity UPLC CSH C18 VanGuard pre-column (5 × 2.1mm, 1.7 μm, Waters), operating at 55 °C. Injection volume of 4 µL sample.

The Acquity UPLC operated at a flow rate of 0.350 µL/min and the mobile phase consisted of acetonitrile—water (60:40; *v*/*v*) with 10 mM ammonium formate (solution A) and isopropanol-acetonitrile (90:10; *v*/*v*) with 10 mM ammonium formate (solution B). Initial conditions started with 40% B, and immediately a linear gradient from 40 to 100% B in 0 to 13 min, the eluent composition returned to the initial conditions in 0.1 min, and the column was equilibrated at the initial conditions for 3 min before the next injection, leading to a total run time of 16 min for each sample. For both positive and negative ionization modes, the same chromatographic conditions were used. Data collection and system control were performed using Waters MassLynx software (version 4.1, Waters Corporation, Milford, MA, USA).

##### Mass Spectrometry Conditions

The detection was performed using a XEVO-G2 Q-TOF (quadrupole time of flight) mass spectrometer (Waters Corporation, Milford, MA, USA) equipped with electrospray ionization (ESI) source. The MS data was acquired in ESI positive and negative ionization modes, in separate runs in full scan mode from 50 to 1200 *m*/*z*, the scan rate of 1 scan/s was used in both. Conditions in positive mode: capillary voltage 3 kV, sampling cone 40 V, extraction cone 4 V, source temperature 120 °C, desolvation temperature 500 °C, cone gas flow rate 50 L/h, desolvation Gas Flow 800 L/h. Conditions in negative mode: capillary voltage 2.5 kV, sampling cone 35 V, extraction cone 4 V, source temperature 120 °C, desolvation temperature 500 °C, cone gas flow rate 50 L/h, desolvation Gas Flow 800 L/h. Data were collected in centroid mode and mass was corrected during acquisition using and Leucine enkephalin ([M + H]^+^ = 556.2771 and [M − H]^−^ = 554.2615) infused via lockspray interface, the scan frequency was 15 s with reference cone voltage of 30 V to ensure accuracy during the MS analysis. MS/MS experiments were performed by ramping the collision energy from 15–60 V on selected masses of interest while all other parameters remained the same.

##### Data Preprocessing

Acquisition of raw UPLC-MS data was performed under MassLynx software (version 4.1, Waters Corporation, Milford, MA, USA). MassLynx was used for visualization and manual processing of data. Mass data were automatically processed by open-source mzMine2 (version 2.23 [28]) using in-house Lipid library and the resulting peak areas were exported into a table in csv format. 

In order to correct for drift originating from long sequence analysis corrections by LOESS logarithm [29] using script for MATLAB were performed. 

### 2.6. Data Analysis

Selected signals observed in the ^1^H NMR spectrum of serum were integrated using an in-house MATLAB script (MathWorks, Inc., Natick, MA, USA). Integration of leucine was performed by Lorentzian deconvolution of overlapping isoleucine (δ 0.93, t) and leucine (δ 0.95, t) signals using Topspin 3.0 (Bruker Biospin). All identified lipids obtained from the LC-MS analyses were integrated and used for data analysis. A linear mixed-effects model was calculated using the lme4 [30], pbkrtest [31] and lsmeans [32] packages in R [33] (https://cran.r-project.org/). The full model included the fixed effects of diet, gender, period (carryover effect), and the diet-gender, diet-period and gender-period interactions, and random effects of subject. The model calculated for the postprandial plasma data included an additional effect of the diet-period-time, diet-gender-time, period-gender-time three-way interactions. It was assumed that data did not contain any 4- or 5-way interactions. For testing statistical significance, the full model was compared to a reduced model without the effects in question, and effects with *p* < 0.05 were considered statistically significant.

## 3. Results

A representative ^1^H NMR spectrum of a serum sample is shown in Figure S2 and the resonance assignments are provided in Table S1. A total of 242 lipid species were identified by LC-MS analyses (Table S2). 

### 3.1. Fasting State

**Figure 1:**
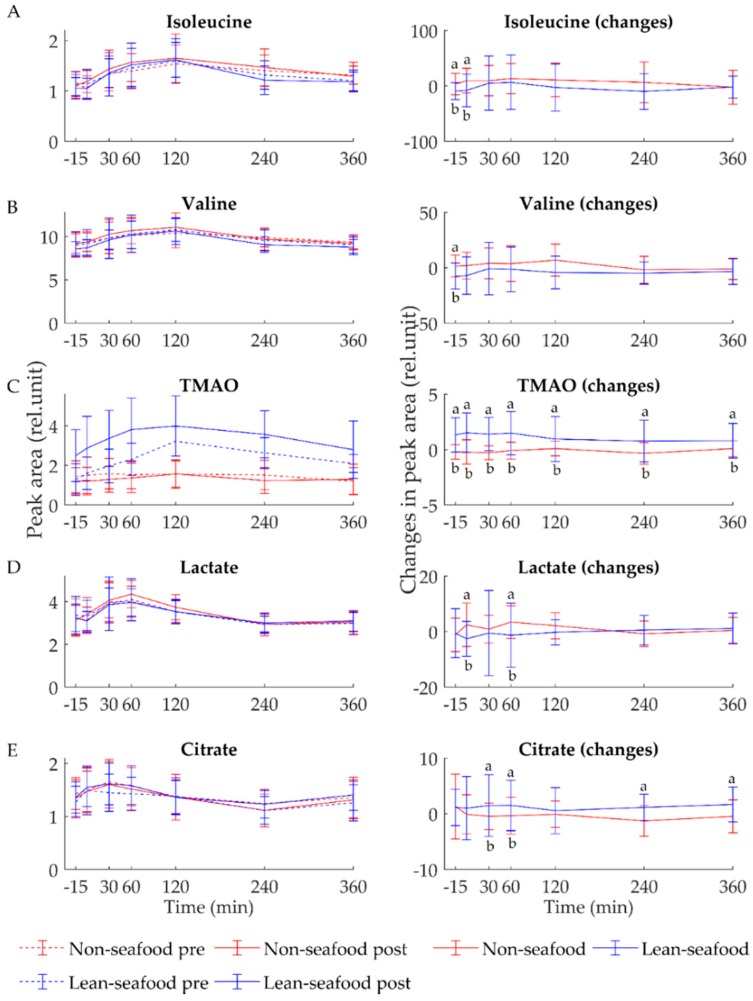
Mean ± SEM of fasting and postprandial (**A**) isoleucine, (**B**) valine, (**C**) trimethylamine N-oxide (TMAO), (**D**) lactate and (**E**) citrate levels observed in serum samples from ^1^H NMR spectroscopy after a 4-week intervention with lean-seafood (blue) and non-seafood diets (red). Baseline values are shown as dotted lines and post-intervention values are shown as continuous lines. Between −15 min (fasting) and 0 min the subjects ingested the test meal. Postprandial blood samples were withdrawn at 30, 60, 120, 240 and 360 min. Changes were calculated as postvalues minus baseline values. Statistical significance was calculated using a linear mixed-effects model. Letters a and b indicates statistical significance of *p* < 0.05.

**Figure 2:**
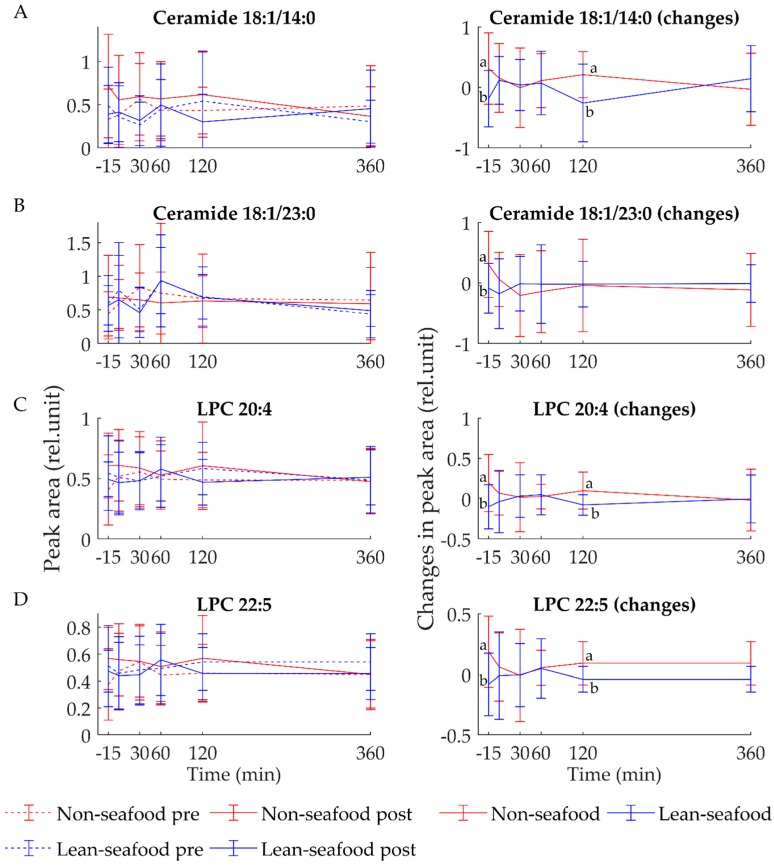
Mean ± SEM of fasting and postprandial (**A**) ceramide 18:1/14:0, (**B**) ceramide 18:1/23:0, (**C**) lysophosphatidylcholine (LPC) 20:4 and (**D**) LPC 22:5 observed in serum samples from LC-MS analyses after a 4-week intervention with lean-seafood (blue) and non-seafood diets (red). Baseline values are shown as dotted lines and post-intervention values are shown as continuous lines. Between −15 min (fasting) and 0 min the subjects ingested the test meal. Changes were calculated as postvalues minus baseline values. Statistical significance was calculated using a linear mixed-effects model. LPC, lysophosphatidylcholines. Letters a and b indicates statistical significance of *p* < 0.05.

The lean-seafood diet, as opposed to the non-seafood diet, significantly reduced fasting serum levels of isoleucine (*p* = 0.04, Figure 1A) and valine (*p* = 0.02, Figure 1B). The same pattern was observed for fasting serum leucine level, even though the changes did not reach statistical significance (*p* = 0.2, Figure S3). Of the 242 lipid species analyzed, a total of 26 lipids were significantly increased in fasting serum after the non-seafood diet period, including ceramide 18:1/14:0 (*p* = 0.01, Figure 2A), 18:1/23:0 (*p* = 0.04, Figure 2B), lysophosphatidylcholines (LPC) 20:4 (*p* = 0.02, Figure 2C) and 22:5 (*p* = 0.02, Figure 2D), free fatty acids (FFA) 20:5 (*p* = 0.04), lysophosphatidylethanolamines (LPE) 18:0 (*p* = 0.02), 18:2 (*p* = 0.02), phosphatidic acids (PA) 32:0 (*p* = 0.03), 38:5 (*p* = 0.02), phosphatidylethanolamines (PE) 18:3/22:1 (*p* = 0.03), 24:4/16:1 (*p* = 0.02), 24:1/18:0 (*p* = 0.04), phosphatidylinositols (PI) 34:0 (*p* = 0.01), 36:0 (*p* = 0.04), 18:0/22:4 (*p* = 0.03), 16:0/22:5 (*p* = 0.03), 40:4 (*p* = 0.01), phosphatidylglycerols (PG) 18:1/16:1 (*p* = 0.01), 22:1/14:0 (*p* = 0.01), 22:2/14:0 (*p* = 0.01), phosphatidylserines (PS) 16:0/16:1 (*p* = 0.02), 20:4/18:0 (*p* = 0.01), 22:4/18:1 (*p* = 0.04) and phosphatidylcholines (PC) 16:0/20:4 (*p* = 0.02), 16:1/20:4 (*p* = 0.05) and 16:2/24:5 (*p* = 0.05) (Table S3). Furthermore, a tendency towards an increased fasting serum levels of ceramide 18:1/20:0 (*p* = 0.08) and 18:1/22:2 (*p* = 0.07) was observed after the non-seafood diet period. 

### 3.2. Postprandial State

Statistical analyses revealed a significant interaction between diet x time x sampling time for the postprandial serum level of TMAO (*p* = 0.008). In fact, the serum level of TMAO was significantly increased at all postprandial time points after lean-seafood intake as compared to after non-seafood intake (Figure 1C). As expected, following a meal intake, the postprandial serum level of absorbed metabolites increases and eventually reaches baseline levels again. This pattern was observed for glucose, lactate, amino acids (isoleucine, valine, leucine, alanine, glycine, tyrosine, histidine, glutamine and phenylalanine) and citrate. Similarly, the opposite pattern was evident for 3-hydroxybutyrate, acetate and formate, for which the metabolite concentration decreased upon diet intake, and then eventually increased during the postprandial period. Thus, the changes of each metabolite were therefore statistically evaluated at each individual postprandial time points. Following the lean-seafood diet, the serum level of lactate was significantly decreased 0 min (*p* = 0.02) and 60 min (*p* < 0.001) postprandial (Figure 1D). Furthermore, the postprandial serum level of citrate was significantly increased 30 min (*p* = 0.03), 60 min (*p* = 0.004), 240 min (*p* = 0.01) and 360 min (*p* = 0.02) after lean-seafood intake (Figure 1E). The serum level of the circulating BCAAs isoleucine, valine and leucine was decreased during the postprandial state after lean-seafood intake. However, only isoleucine reached statistical significance 0 min postprandial (*p* = 0.02, Figure 1A), and a tendency towards a decreased level of valine was observed 0 min postprandial (*p* = 0.06, Figure 1B). Statistical analyses did not reveal any significant differences for serum glucose, formate, tyrosine, acetate, glutamine, phenylalanine, histidine, glycine, alanine or leucine at any of the postprandial time points (Figure S3). The postprandial changes in the serum level of the lipid species revealed that the non-seafood diet significantly increased the serum level of triacylglycerol (TAG) 48:2 at time 120 min (*p* = 0.04) and 360 min (*p* = 0.04) (Table S3). Furthermore, the serum level of TAG 48:1 was also significantly increased at time 360 min (*p* = 0.02), and a trend towards an increased serum level of 48:1 was observed at 120 min after the non-seafood diet period (*p* = 0.08, Table S3). Furthermore, 120 min postprandial the non-seafood diet significantly increased level of ceramide 18:1/14:0 (*p* = 0.01, Figure 2A), LPC 20:4 (*p* = 0.01, Figure 2C) and 22:5 (*p* = 0.03, Figure 2D), LPE 18:0 (*p* = 0.009), PA 34:2 (*p* = 0.01), 36:1 (*p* = 0.01), 36:2 (*p* = 0.02), 36:1 (*p* = 0.01), PE 34:0 (*p* = 0.01), 34:1 (*p* = 0.004), 34:2 (*p* = 0.03), 36:3 (*p* = 0.01), 40:4 (*p* = 0.005), 40:5 (*p* = 0.04), PE 40:4 (*p* = 0.005), PE 40:5 (*p* = 0.04), PC 34:3 (*p* = 0.02), PC 36:4 (*p* = 0.007), PC 36:5 (*p* = 0.004), PC 38:5 (*p* = 0.005), PC 40:5 (*p* = 0.04) and PC 40:8 (*p* = 0.04) (Table S3). 

### 3.3. Dietary Amino Acid Composition

We analyzed the diets using ^1^H NMR spectroscopy in order to evaluate the contribution of specific dietary compounds to changes in serum metabolites. The diet analysis revealed no significant difference in the concentrations of isoleucine (non-seafood diet = 0.02 mg/g dry diet; lean-seafood diet = 0.01 mg/g dry diet, *p* = 0.13) and valine (non-seafood diet = 0.04 mg/g dry diet; lean-seafood diet = 0.03, *p* = 0.25) between the diets.

## 4. Discussion

The present study shows that 4 weeks of dietary intervention with lean-seafood or non-seafood diets differentially modulate serum metabolites in healthy adults. The changes indicate that intake of lean-seafood, as compared to intake of non-seafood, influence markers of energy metabolism. The lean-seafood diet period decreased fasting circulating isoleucine and valine, whereas the non-seafood diet increased the fasting level of certain ceramides and lysophosphatidylcholine lipid species. Furthermore, relative to the non-seafood diet period, fasting and postprandial TMAO levels were significantly increased following the lean-seafood diet period, which is of interest as studies have proposed a link between high circulating TMAO levels and increased CVD risk [34,35]. 

### 4.1. Lean-Seafood Intake Modulate Markers of Energy Metabolism

**Figure 3:**
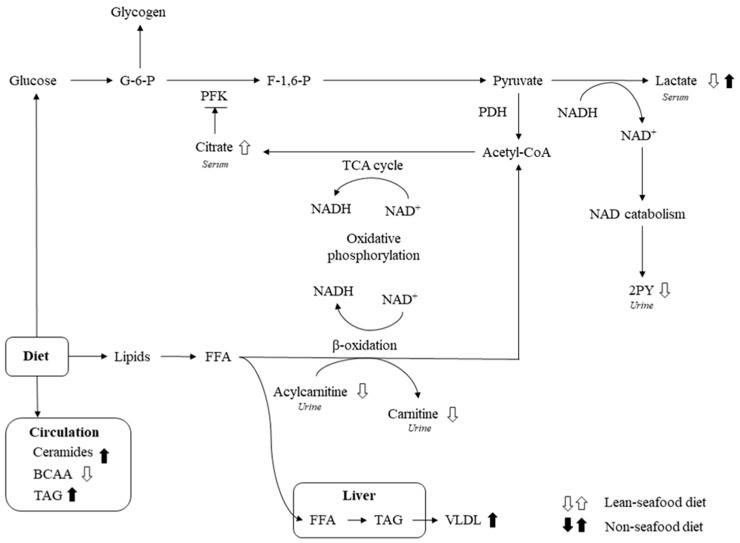
Schematic summary of how 4-weeks intervention with lean-seafood and non-seafood diets affect mitochondrial fuel selection. PDH, pyruvate dehydrogenase; PFK, phosphofructokinase; G-6-P, glucose-6-phosphat; F-1,6-P, fructose-1,6-biphosphat; 2PY, *N*-methyl2-pyridone-5-carboxamide; TAG, triacylglycerol; VLDL, very-low-density lipoprotein; FFA, free fatty acids; TCA, tricarboxylic acid.

Glucose and fatty acids are the major substrates for generation of cellular energy, and mitochondrial status has been shown to affect fuel selection [36]. Previously, we showed that the lean-seafood diet period reduced the urinary content of *N*-methyl-2-pyridone-5-carboxamide (2PY), acylcarnitine and carnitine, indicative of an improved mitochondrial function [25]. Intriguingly, elevated urinary excretion of 2PY has been suggested as a biomarker of type 2 diabetes progression [37], and reduced circulating acylcarnitines may reflect maintenance of sufficient mitochondria fatty acid oxidation capacity and preservation of insulin sensitivity [38,39]. In support of this, we found an elevated postprandial lactate level after the non-seafood diet period. The variations in serum lactate following lean-seafood and non-seafood intake has previously been published by Aadland et al. [24], in which serum lactate levels were measured by standard biochemical assay. The NMR spectroscopic analyses of serum lactate verified this finding. The underlying mechanism for the increased lactate level after non-seafood intake could be impaired insulin sensitivity in peripheral tissue, which reduces the capacity to store ingested glucose as glycogen [40,41], and consequently more of the ingested carbohydrate is converted to lactate [40], which may be used in hepatic lipogenesis resulting in dyslipidemia with elevated TAG and reduced high density lipoprotein (HDL) [41] (Figure 3). To support this notion, we previously reported that the lean-seafood diet reduced fasting and postprandial TAG, and prevented higher total- to HDL cholesterol ratio relative to the non-seafood diet period [23]. Furthermore, in the current study we observed an elevated postprandial citrate level after the lean-seafood diet period, which might indicate elevated TCA cycle flux. Increased citrate levels are known to inhibit phosphofructokinase, the most prominent regulatory enzyme in glycolysis [42], which suggest that high citrate levels spare glucose and reroute it for glycogen storage. Moreover, elevated plasma and urine citrate levels have been inversely associated with hepatic lipid levels [43]. In type 2 diabetic subjects, muscle glycogen synthesis is reduced as a result of an impaired insulin-stimulated glucose transport [44,45]. Thus, the present findings may indicate that lean-seafood intake imposes effects likely to reflect a shift in energy metabolism towards improved mitochondrial fatty acid oxidation.

### 4.2. Lean-Seafood Intake Decreases Circulating BCAAs

Certain amino acids have been associated with conditions such as insulin resistance and type 2 diabetes, and studies have shown that serum levels of circulating BCAAs including isoleucine, valine, and leucine are significantly increased in obese and diabetic subjects compared to lean controls [6,46]. In the current study, a significantly reduced level of isoleucine and valine was observed after the lean-seafood intake. Analyses of the meal compositions showed a slightly lower content of isoleucine and valine in the seafood diets compared with the non-seafood diets, however, the differences were not significant and can most likely not explain the differences observed in serum in the fasting state. Thus, the mechanisms underlying these observations are not clear, however, it can be speculated that the observed variations in fasting isoleucine and valine are direct endogenous effects of the intervention diets on the host’s own metabolic pathways. Recently, in a human study, established to identify pathways related to insulin resistance, the combination of gene expression and metabolomics revealed perturbed BCAA catabolism and fatty acid oxidation in skeletal muscle from insulin-resistant human subjects [47]. Furthermore, in a mouse model, the impact of adipose tissue BCAA oxidation to modulate circulating BCAAs has been demonstrated [48]. Thus, a possible link between intake of lean-seafood and an improved BCAA oxidation might exist. Furthermore, a pilot, short-term dietary intervention study, conducted in healthy men using two balanced diets with high or low BCAA content, showed that fasting BCAA levels were only modestly influenced by dietary BCAA manipulation [49] and points to the fact that fasting BCAA levels are not affected by dietary factors alone. However, it cannot be ruled out that a diet-induced modulation of the gut microbiome and activity, with a resultant change in the gut-related production of isoleucine and valine, may have caused or contributed to the variation in circulating BCAAs. Pedersen et al. recently showed that in insulin-resistant non-diabetic subjects, an increased level of circulating BCAAs were correlated with a gut microbiome characterized by an enriched biosynthetic potential of BCAAs and depletion of genes encoding bacterial BCAA uptake [50]. However, the current study did not establish a link between variation in circulating isoleucine and valine and changes in gut microbiome-associated biosynthetic BCAA potential. Previously, we correlated fecal metabolite levels with the gut microbiota, and observed a significant correlations between fecal isoleucine (R = 0.28, *p* = 0.02) and *Anaerotruncus* [26]. In addition, a trend towards a positive correlation was observed between circulating isoleucine and *Anaerotruncus* (R = 0.24, *p* = 0.05, Table S4). These findings indicate a potential role of *Anaerotruncus* in isoleucine production. Thus, our findings show that lean-seafood intake reduce fasting levels of circulating isoleucine and valine compared to non-seafood intake. However, the underlying mechanisms for these observations could not be specifically identified, and in order to establish a causal association between *Anaerotruncus* and circulating isoleucine, and the potential role of lean-seafood and non-seafood proteins to modulate the gut microbiota-associated BCAA biosynthetic potential, further studies are warranted. 

### 4.3. Non-Seafood Intake Increases Circulating Ceramides and Lysophosphatidylcholine Species

In type 2 diabetic patients, the levels of circulating ceramides are markedly increased [51] and studies in rodents and human muscle cells indicate that blocking ceramide synthesis can prevent the development of insulin resistance [52,53]. Furthermore, at the cellular level, ceramides have been shown to impair insulin signaling and intracellular handling of glucose and lipids with resultant deleterious effects on cellular metabolism [54,55]. However, the associations between ceramide levels and insulin sensitivity and the risk of type 2 diabetes are not consistent [56,57]. In the current study, we found a higher fasting level of certain ceramide species (ceramide 18:1/14:0 and 18:1/23:0) after the non-seafood diet period, which might indicate that 4 weeks of non-seafood dietary intervention influences serum ceramide levels. One of the main pathways by which ceramides can be synthesized are through de novo synthesis. Several stress stimuli such as inflammatory mediators, chemotherapeutics and oxidative stress can induce ceramide production and lead to accumulation of ceramide species [58]. In the study by Haus et al. a higher fasting level of ceramide 18:1/20:0 and total ceramides were observed in type 2 diabetic subjects compared to lean, healthy individuals [51], and in a mice study, high-fat feeding gave rise to increased levels of liver ceramides, including 18:1/20:0 [59]. In addition, ceramide 18:1/16:0 have also been identified as a key ceramide that negatively regulates insulin sensitivity and fatty acid oxidation in obesity [60].

Thus, a potential role of circulating ceramides to influence insulin sensitivity and action may exist, yet, several ceramides seems to be involved in these mechanisms. Accordingly, further studies are warranted in order to evaluate the physiological role of individual ceramides subspecies in relation to insulin resistance and type 2 diabetes. Another interesting finding in our study is the increased level of fasting lysophosphatidylcholine (LPC) species detected after the non-seafood diet period, as these lipid species may play a role in atherosclerosis and inflammation [61,62]. Furthermore, in a recent longitudinal cohort study, several LPCs were downregulated in subjects who regressed from a pre-diabetic state to normal glucose regulation [63]. In contrast, lower levels of LPC lipid species have also been observed in insulin-resistant humans [64]. Thus, in insulin-resistant subjects, changes of circulating LPC lipid species reveal conflicting results, and further studies are warranted in order to evaluate the physiological relevance of changes in circulating LPC lipid species. 

### 4.4. Lean-Seafood Intake Increases Circulating TMAO Levels but Decreases Other Risk Markers of CVD

During recent years, TMAO has received considerable attention due to its proposed association with atherosclerosis and CVD [34,35]. TMA, a metabolite derived from bacterial metabolism of choline and carnitine, which is abundantly present in eggs and beef, is absorbed into the circulation and subsequently oxidized in the liver with resultant increases in plasma TMAO levels. Recently, chronic choline feeding of atherosclerosis-prone mice promoted vascular inflammation, and acute TMAO injection activated inflammatory signaling and gene expression pathways [65]. These observations were repeated in primary human aortic endothelial cells and vascular smooth muscle cells, and TMAO were found to enhance the endothelial recruitment of activated leukocytes [65], a marker of pro-inflammatory conditions [66]. Furthermore, dietary supplementation of mice with choline and TMAO promoted macrophage foam cell formation, an early hallmark of the atherosclerotic process [34]. Based on these results, it has been proposed that high plasma TMAO levels might induce an atherosclerotic phenotype. Intriguingly, fish and seafood contain considerable amounts of free TMAO, and consumption of cod fillet has been shown to increase circulating TMAO to a greater extent than intake of eggs or beef [67]. Intake of fish and seafood is considered to have a beneficial effect on heath [68,69]. In addition, a reduced atherosclerotic plaque burden had been observed in *apoE*^−/−^ deficient mice fed a Western type diet containing a combination of cod and scallops as compared to chicken fed mice [70], and a recent cross-sectional study showed that dietary inclusion of lean-fish, rich in TMAO, is associated with a lower risk of developing the metabolic syndrome [71]. Thus, consumption of beef, eggs, fish and seafood gives rise to increased circulating TMAO levels. However, a paradox seems to arise as intake of choline- and carnitine-rich foods have been associated with increased risk of atherosclerosis and CVD, whereas intake of fish and seafood reduces this risk [23,72]. In the current study, increased serum level of TMAO was observed at 30 min postprandial. The finding suggests that free TMAO is readily absorbed from the lean-seafood diet, which is consistent with findings obtained by Cho et al. [67]. These observations indicate that circulating TMAO may merely be an exposure marker of fish and seafood intake. Previously, we showed that 4-weeks intervention with lean-seafood reduced fasting and postprandial serum TAG levels [23], beneficially modulated risk factors for type 2 diabetes [24] and reduced urinary markers of mitochondrial lipid and energy metabolism [25]. Thus, the present study does not support an association between high circulating TMAO levels and increased risk of atherosclerosis and CVD. On the contrary, the present findings support the notion of TMAO as an exposure marker of dietary consumption of fish [67,73], and that dietary inclusion of lean-seafood is associated with beneficial effects on CVD risk factors [21,74,75]. 

### 4.5. Dietary Exposure Markers

In the current study, to balance for the marine n-3 fatty acids present in the lean-seafood diets, cod liver oil was added to all non-seafood dinner meals prior to serving [23]. The bioavailability of n-3 fatty acids might be influenced by the form of delivery [76]. In lean seafood, n-3 fatty acids are primarily bound as phospholipids, whereas in fish oil n-3 fatty acids are primarily bound in TAG form [77,78]. These structural differences in form of delivery might influence the bioavailability of n-3 fatty acids and the resultant circulating serum levels [79]. Thus, in the current study, the increased level of polyunsaturated fatty acid (PUFA) lipid species observed after the non-seafood diet period is anticipated, at least in part, to be a direct dietary exposure marker. Furthermore, even though the level of total PUFA was balanced between the experimental diets, the level of the individual PUFAs were not equal between the diets [23], which also could be a contributing factor influencing the present findings. 

## 5. Conclusions

In conclusion, the current study reveals that 4 weeks of intervention with lean-seafood and non-seafood diets that varied mainly in protein source differentially modulates fasting and postprandial serum metabolites in healthy human subjects. The lean-seafood diet period, as opposed to the non-seafood diet period, reduced serum lactate and increased citrate levels, which might be indicative of a shift in energy metabolism. Furthermore, intake of lean-seafood reduced circulating isoleucine and valine, whereas intake of non-seafood increased circulating level of some ceramide and LPC species. Also, the lean-seafood diet gave rise to significantly higher fasting and postprandial TMAO serum levels, which is expected to originate from the TMAO content of the lean-seafood diet. Thus, our data show that lean-seafood and non-seafood protein differentially modulate serum metabolites in healthy humans. Further studies are needed to clarify whether these changes are of physiological relevance.

## Supplementary Materials

The following are available online at http://www.mdpi.com/2072-6643/10/5/598/s1, Figure S1: Experimental study design; Figure S2: ^1^H NMR spectrum of human serum sample; Figure S3: Mean ± SEM fasting and postprandial metabolites levels observed in serum samples from ^1^H NMR spectroscopy; Table S1: ^1^H NMR resonance assignments; Table S2: LC-MS assignments; Table S3: Fasting and postprandial lipid species; Table S4: Pearson correlations between serum metabolites and bacterial16S rRNA sequencing information.
