# Supplementary material for: The Effect of Lean-Seafood and Non-Seafood Diets on Fasting and Postprandial Serum Metabolites and Lipid Species: Results from a Randomized Crossover Intervention Study in Healthy Adults
Source: Nutrients. 2018 May 11;10(5):598. doi: 10.3390/nu10050598 (PMC5986478; doi:10.3390/nu10050598)
Supplement: Supplementary file 1 [file nutrients-10-00598-s001.zip › Table S1.docx]

**Table S1.** Assignment of resonances obtained from ^1^H NMR spectra of serum from healthy subjects after 4-weeks intervention with lean-seafood and non-seafood diets.

| **No.** | **Metabolites** | **δ ^1^H** | **Moieties 1H** | **δ ^13^C** |
| --- | --- | --- | --- | --- |
| 1 | Acetate | 1.91 (s) | CH_3_ | - |
| 2 | Acetoacetic acid | 2.27 (s) | CH_3_ | - |
| 3 | Acetone | 2.22 (s) | CH_2_ | - |
| 4 | Alanine | 1.47 (d) | CH_3_ | 19.07 |
| 5 | Citrate | 2.52 (d); 2.69 (d) | ½CH_2_; ½CH_2_ | - |
| 6 | Creatine | 3.92 (s); | CH_2_ | - |
| 7 | Creatinine | 3.03 (s), 4.04 (s) | CH_3_; CH_2_ | 59.24 |
| 8 | Formate | 8.45 (s) | CH_3_ | - |
| 9 | α-Glucose | 3.53 (dd); 3.70 (t); 3.83 (m); 5.23 (d) | CH3; CH4/CH11; CH11/CH6; CH2 | 74.34; 75.56; 63.44; 94.95 |
| 10 | β-Glucose | 3.24 (dd); 3.40 (t), 3.45 (m); 3.48 (t), 3.71 (dd), 3.89 (dd) | CH3; CH5; CH6; CH3; CH6 | 77.02;72.41; 78.72; 72.52;75.52; 63.28 |
| 11 | Glutamine | 2.12 (m); 2.45 (m) | CH_2_; CH_2_; | 29.47; 33.77 |
| 12 | Glycine | 3.55 (s) | CH_2_ | 44.43 |
| 13 | Histidine | 7.04 (s); 7.74 (s) | CH; CH | - |
| 14 | 3-hydroxybutyrate | 1.19 (d); 2.30 (d); 2.39 (d); 4.14 (m) | CH_3_; CH_2_; CH_2_; CH | - |
| 15 | Isoleucine | 0.93 (t); 1.00 (d) | β-CH_3_; | - |
| 16 | Lactate | 1.32 (d); 4.10 (q) | CH_3_; CH | 22.88; 71.31 |
| 17 | Leucine | 0.95 (t), 1.72 (m) | δ-CH_3_; CH_2_ | 24.79; 42.91 |
| 18 | Lipid | 0.85 (m); 1.26 (m); 1.99 (m) | CH_3_(CH_2_)_n_; (CH_2_)_n_; CH_2_C=C | - |
| 19 | Lysine^a^ | 1.71 (m); 1.89 (m) | CH_2_; CH_2_ | - |
| 20 | N-acetyl glycoproteins | 2.03 (s) | CH_3_ | - |
| 21 | TMAO | 3.25 (s) | CH_3_ | 62.3 |
| 22 | Phenylalanine | 3.98 (dd); 7.31 (d); 7.41 (m) | CH2/CH6; CH3/CH5 | - |
| 23 | Tyrosine | 6.89 (d); 7.19 (d) | CH3/CH5; CH2/CH6 | 118.8;- |
| 24 | Unsaturated lipid | 5.28 (m) | -CH=CH- | - |
| 25 | Valine | 0.98 (d); 1.03 (d); 3.60 (d) | CH_3_; CH_3_; CH | 19.41; 20.73; - |

TMAO; trimethylamine N-oxide
^a^ Tentatively assigned
