# Supplementary material for: The Effect of Lean-Seafood and Non-Seafood Diets on Fasting and Postprandial Serum Metabolites and Lipid Species: Results from a Randomized Crossover Intervention Study in Healthy Adults
Source: Nutrients. 2018 May 11;10(5):598. doi: 10.3390/nu10050598 (PMC5986478; doi:10.3390/nu10050598)
Supplement: Supplementary file 1 [file nutrients-10-00598-s001.zip › Table S3.docx]

**Table S3.** Significantly increased lipid species observed in the fasting and postprandial state after 4-weeks of non-seafood intervention. Changes were calculated as postvalues minus baseline values. Statistical significance was calculated using a linear mixed-effects model.

| **Lipid** | **-15 min** | **0 min** | **30 min** | **60 min** | **120 min** | **360 min** |
| --- | --- | --- | --- | --- | --- | --- |
| FFA | 20:5 | 18:0 20:0 20:4 |  |  |  |  |
| LPE | 18:0/0:0 18:2/0:0 |  |  |  | 18:0/0:0 |  |
| Cer |  | 42:0 |  |  |  |  |
| PA | 32:0  38:5 |  |  |  | 34:2 36:1  36:2 |  |
| PE | 18:3/22:1 24:4/16:1 24:1/18:0 |  |  |  | 34:0 34:1  34:2  36:3 18:3/22:1  24:4/16:1 |  |
| PI | 34:0 36:0 16:0/22:4 16:0/22:5 18:0/22:4 | 38:6 |  | 16:0/22:4 | 36:3 |  |
| PG | 18:1/16:0 18:1/16:1 22:1/14:0 22:2/14:0 |  | 36:4 |  |  |  |
| PS | 16:0/16:1 20:4/18:0 22:4/18:1 |  | 40:4 | 40:6 | 36:2 38:5 |  |
| PC | 16:0/20:4 16:1/20:4 16:2/24:5 | 34:0 |  |  | 34:3 16:0/20:4 16:1/20:4 38:5  40:5  40:8 |  |
| TAG |  |  |  |  | 48:2 50:1 50:2  52:1  54:3 | 48:2 48:1 |
