# Supplementary figures and images for: The Effect of Lean-Seafood and Non-Seafood Diets on Fasting and Postprandial Serum Metabolites and Lipid Species: Results from a Randomized Crossover Intervention Study in Healthy Adults
Source: Nutrients. 2018 May 11;10(5):598. doi: 10.3390/nu10050598 (PMC5986478; doi:10.3390/nu10050598)

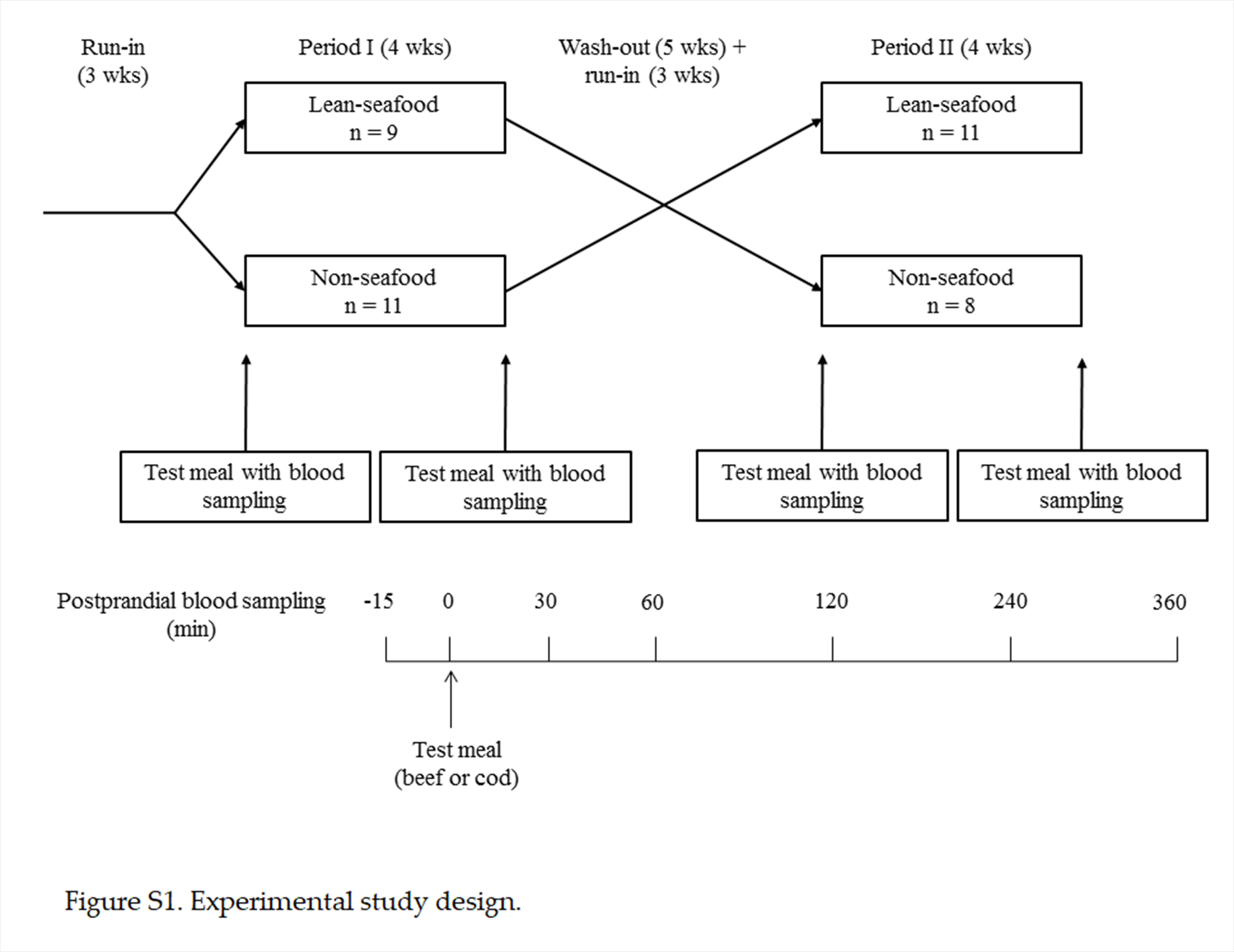

Supplement: Supplementary file 1 [file nutrients-10-00598-s001.zip › Figure S1.tif]

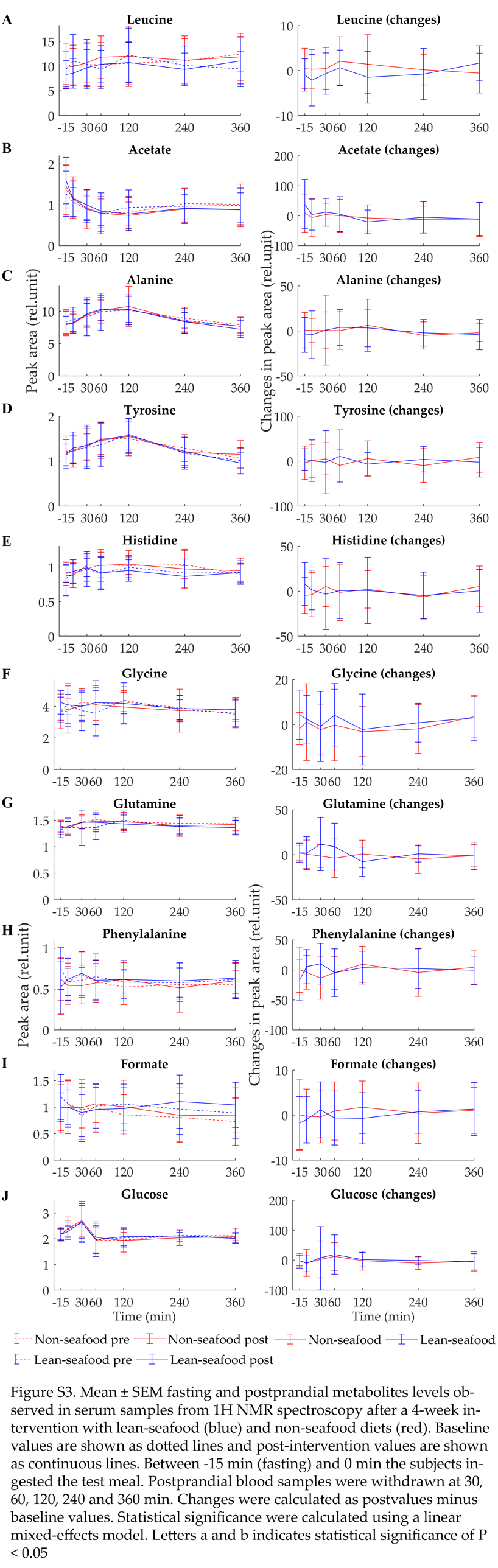

Supplement: Supplementary file 1 [file nutrients-10-00598-s001.zip › Figure S3.tif]
